# Case Report: Combined Intravenous Infusion and Local Injection of CAR-T Cells Induced Remission in a Relapsed Diffuse Large B-Cell Lymphoma Patient

**DOI:** 10.3389/fimmu.2021.665230

**Published:** 2021-04-19

**Authors:** Linhui Hu, Fan Wu, Huiping Wang, Weiwei Zhu, Juan Wang, Fengxiang Yu, Zhimin Zhai

**Affiliations:** ^1^ Department of Hematology/Hematological Lab, The Second Hospital of Anhui Medical University, Hefei, China; ^2^ Jiangsu Tuohong Kangheng Pharmaceutical Co. Ltd, Nanjing, China

**Keywords:** chimeric antigen receptor T cell therapy, local injection, diffuse large B-cell lymphoma, extranodal disease, case report

## Abstract

Relapsed diffuse large B-cell lymphoma (DLBCL) is a disease with a poor prognosis. Recent clinical trials results showed chimeric antigen receptor (CAR) T cell therapy has a promising role in treating relapsed DLBCL. Unfortunately, patients with extranodal lesions respond poorly to CAR-T cells administered intravenously. Herein, we evaluated the efficacy and safety of a new treatment strategy of CAR-T cells, combining intravenous infusion with local injection of CAR-T cells, in a relapsed DLBCL patient with extranodal lesions. The patient achieved durable remission and without severe adverse effects after CAR-T cells treatment. During the follow-up period of one year, the patient remained in good condition. In conclusion, combining intravenous injection with a local injection for CAR-T cell is a feasible strategy for relapsed DLBCL patients with extranodal lesions.

## Introduction

Diffuse large B-cell lymphoma is the most common type of adult Non-Hodgkin lymphoma; although 60–70% of patients are sensitive to R-CHOP, 30–40% of patients will relapse or become refractory to R-CHOP within the first 2–3 years (1). Relapsed/refractory diffuse large B-cell lymphoma is a disease with a poor prognosis (2), with 3-year overall survival of 49% after salvage treatment (3). Recent clinical trial results showed chimeric antigen receptor (CAR) T-cell therapy has a promising efficacy in patients with leukemia (4) and lymphoma (5). In CAR-T cell treated relapsed/refractory DLBCL patients, the complete response rate ranged from 40–58% (5). Undoubtedly, CAR-T cell therapy offers a new therapeutic option for relapsed/refractory DLBCL patients.

Recently, the impact of extranodal lesions on CAR-T cell therapy has been evaluated. Extranodal lesions present a poor response than lymph nodes in R/R Hodgkin’s lymphoma patients (6), and extranodal lesions are a poor indicator of early progression in R/R DLBCL patients (7). What’s more, soft tissue infiltration correlated with adverse prognosis in R/R DLBLC patients (8). Clinically, approximately 40% of DLBCL patients present with extranodal lesions. Thus, new treatment strategies of CAR-T cell therapy need to be developed to improve treatment outcomes and decrease the relapse rate for R/R DLBCL patients with extranodal lesions.

In lymphoma patients, CAR-T cells are given as an intravenous infusion, and it moves through the blood to the site of the lesions, but in solid tumors, the infused of CAR-T cells may not reach (“barrier effect”) the extranodal lesions in DLBCL patients (9). Herein, we report a new treatment strategy for CAR-T cell therapy in a relapsed DLBCL patient with extranodal lesions. We combined intravenous infusion with local injection of CAR-T cells and then evaluated its efficacy and safety. Our result may shed some light on the treatment strategy of CAR-T cell therapy.

## Case Presentation

A 60-year-old female patient was admitted to our hospital and enrolled in our clinical trial for relapsed diffuse large B cell lymphoma. In 2014, the patient complained of a mass in her right breast; subsequently, her pathological diagnosis was DLBCL. The detail immunohistochemical findings were as follows: CD5(+), CD3(+), CD20(+), CD79a(+), CD10(scattered weak+), Mum-1(+), Bcl-2(+), Ki-67(>80%), and Bcl-6(+). The patient refused medical treatment for personal reasons.

Approximately 4 years later (2018), the disease progressed; she complained of a new mass that emerged in her right forearm. Further PET/CT examination indicated increased metabolism in the ethmoid sinus, nasal cavity, nasopharynx, bilateral mammary glands, chest wall on the left side, right back, waist, and subcutaneous of both upper limbs. A biopsy of right forearm mass was performed, and immunohistochemical findings were as follows: CD20(+), PAX5(+), CD10(−), Mum-1(+), bcl-2(+), bcl-6(+), cyclinD1(−), TDT(−), Ki-67(60%). Based on these findings, she was diagnosed with DLBCL. Then, she received five cycles of induction therapy of rituximab, cyclophosphamide, doxorubicin, vincristine, and prednisone (R-CHOP) and achieved complete remission. In the last course of induction therapy, the patient had a severe lung infection; therefore, she refused to receive further medical treatment.

**Figure 1:**
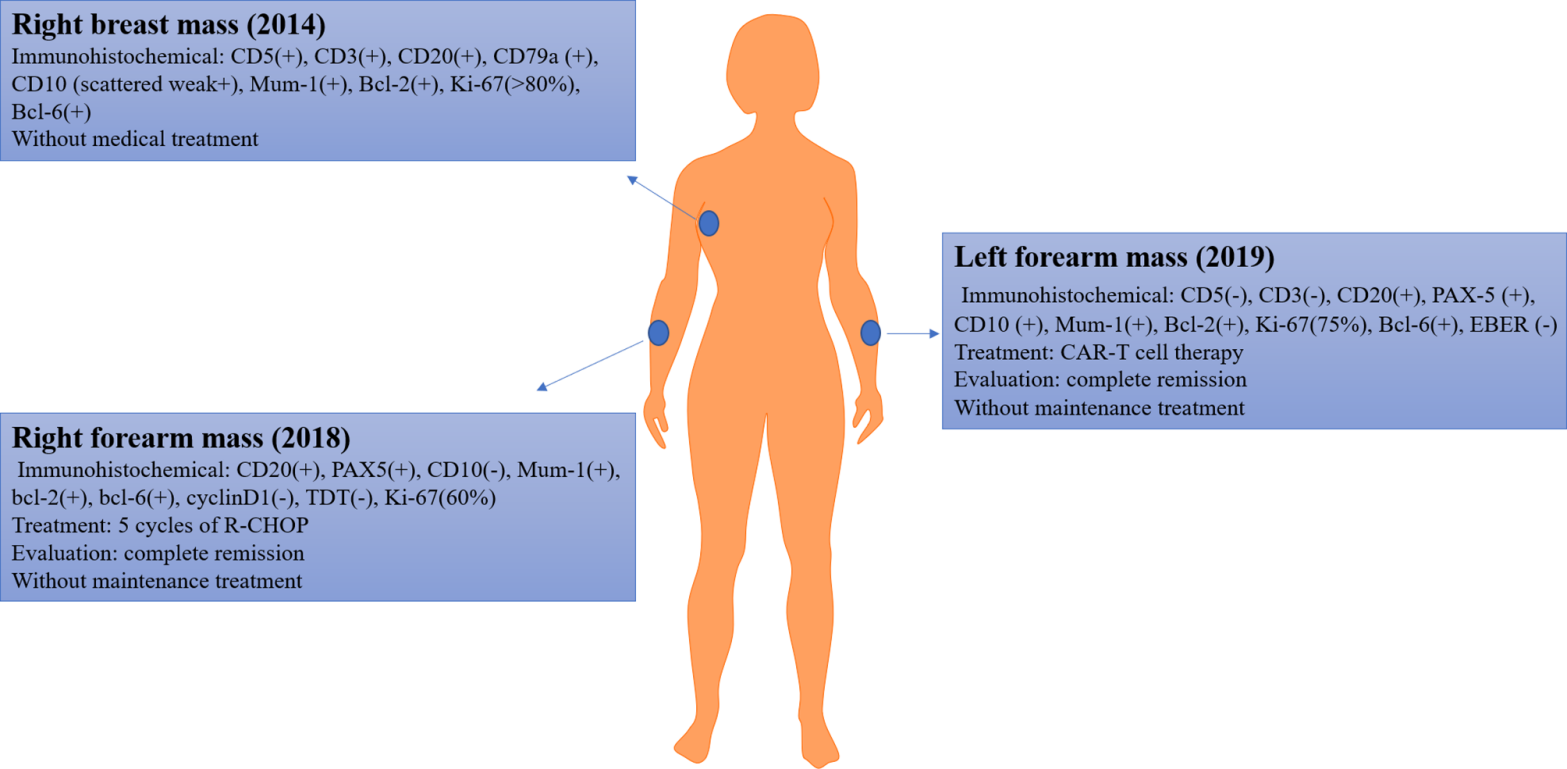
The disease history of the patient. The blue circle indicates the lesion.

In October 2019, a new mass emerged in her left forearm (size: 3 cm ∗ 3 cm ∗ 1 cm), with a scab; without pain, pruritus, or secreta in her left forearm; and without fever, hidrosis, or weakness. The ultrasound indicated enlargement of superficial lymph nodes (cervical, axillary, and inguinal lymph nodes). To identify the pathologic type of this new mass, a biopsy was performed and immunohistochemical findings were as follows: CD5(−), CD3(−), CD20(+), PAX-5(+), CD10(+), Mum-1(+), Bcl-2(+), Ki-67(75%), Bcl-6(+), EBER(−). Based on those findings, the patient was diagnosed with relapsed DLBCL (Ann Arbor stage IV). A brief introduction of disease history was presented in **Figure 1**.

According to the NCCN guideline (10), we suggested the patient enroll in a clinical trial, and the patient and her families were urged to participate in an ongoing clinical trial of CAR-T cell therapy. Therefore, CD19 CAR-T cell therapy was administered.

### CAR-T Cell Therapy Procedures

Lymphocytes were isolated from peripheral blood and T cells used to generate CD19 CAR-T cells. The lenti-CD19-2^nd^ CAR was provided by Jiangsu Tuohong Kangheng Pharmaceutical Co. Ltd (Nanjing, China). The CAR included a murine anti-CD19 single-chain variable fragment, 4-1BB costimulatory domain, and CD3ζ T cell activation domain. We followed a published CAR-T cell manufacturing process protocol (11).

The patient received conditioning chemotherapy (cyclophosphamide 750 mg/m^2^ for 3 days), followed by CD19 CAR-T cells therapy (cell vitality: 95%, CAR19-positive rate: 47%, cell number: a total of 6.7 × 107 cells (1 × 10^6^ cells/kg), CD4/CD8 T cell ratio: 7.15).

**Figure 2:**
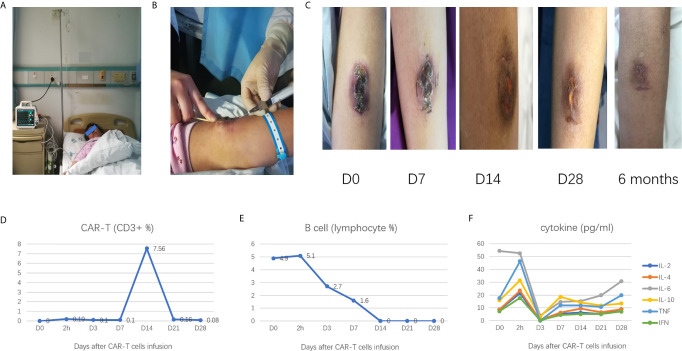
**(A)** intravenous infusion of CAR-T cells; **(B)** local injection of CAR-T cells; **(C)** the changes of mass after CAR-T cell treatment; **(D)** the CAR-T cell kinetics during the treatment process; **(E)** B cell depletion during the treatment process; **(F)** Change in cytokines levels during the treatment process.

In CAR-T cells treated patients, the number of CAR-T cells infiltrating lesions was reported as a more important indicator of effectiveness than the number of CAR-T cells in blood (6). To increase the number of CAR-T cells in local lesions to enhance the efficacy, we used a new strategy that combined intravenous infusion and local injection of CAR-T cells (**Figures 2A, B**).

6.53 × 10^7^ cells were mixed with 100 ml of saline for the intravenous infusion, and 1.67 × 10^6^ cells were mixed with 3 ml of saline for the local injection. A total of six sites were selected for the local injection (four sites at the base of the mass and two sites at the top of the mass), each of the six sites were injected with 0.5 ml CAR-T cells (**Figure 2B**).

### Response to CAR-T Cell Therapy

On day 7, after the CAR-T cell therapy, the mass became flattened, and the scab loosened; on day 14, the mass had a clear reduction in size, the scab fell off, and the median surface was slightly swollen, on day 28, the mass had largely disappeared (**Figure 2C**).

PET/CT examination revealed that the skin on the left forearm’s radial side was slightly thickened and FDG metabolism slightly increased, indicating the patient had achieved a complete remission (CR).

### CAR-T Cell Kinetics

About 2 h after the CAR-T cells therapy, the CAR-T cell was detectable in the peripheral blood and reached its highest peak on day 14; it was almost undetectable on day 28 (**Figure 2D**). Besides, flow cytometry showed B cells in the peripheral blood had decreased from day 1 of CAR-T cells administered level to undetectable level on day 14; the B cells remained unrecovered on day 28 (**Figure 2E**).

### Adverse Effects

The patient did not complain of any pain and discomfort during the process of treatment. There was no liver and kidney function damage or decreased in the hemogram (data not shown). The serum levels of interleukin-6, interleukin-8, interleukin-10, and tumor necrosis factor α slightly fluctuated in the normal range (**Figure 2F**). No signs of severe cytokine-release syndrome (CRS) or tumor lysis syndrome, or neurologic events were observed.

### Follow Up

After CAR-T cell therapy, the maintenance treatment was suggested, but the patient refused to receive further medical treatment. We advised the patient to return for a follow-up visit within six months, and six months after CAR-T cell therapy, the patient had no discomfort, the mass had disappeared, and the newly formed skin was flat (**Figure 2C**). B cells were detectable in the peripheral blood (7.4% of total lymphocyte). One year after CAR-T cells therapy, the patient remained in good condition.

## Discussion

In this study, we reported a new treatment strategy of CAR-T cell therapy that combines intravenous infusion with local injection in a relapsed DLBCL patient with extranodal lesions. The patient achieved durable remission without severe adverse effects. To our best knowledge, this is the first case report on the efficacy of treatment with combined intravenous infusion and local injection of CAR-T cells in a relapsed DLBCL patient with extranodal lesions.

The patient was initially diagnosed with DLBCL in 2014, and the lymphoma progressed in 2018 and 2019, respectively. The disease behavior for the DLBCL is unusual, that lymphoma stays indolent for four years. In spite of this, all three immunohistochemical findings supported the diagnosis of DLBCL, therefore, the patient was diagnosed with relapsed DLBCL in our hospital. The treatment guideline of DLBCL usually recommends clinical trials for relapsed or refractory patients (3). Consistent with the guideline, the patient was enrolled in CAR-T cell therapy’s clinical trial after relapse from R-CHOP. In CAR-T cells treated lymphoma patients, extranodal lesions generally correlate with poor response and poor outcome (6–8). The reason may be the relatively less blood supply and the barrier effect of extranodal lesions leading to a low number of CAR-T cells in local lesions. It may be difficult to get adequate quantities of CAR-T cells trafficked to the forearm lesion sites to have a therapeutic effect with intravenous injection alone. To enhance CAR-T cell efficacy in the local lesion, we combined intravenous injection with local injection in this patient. Besides, in solid tumors, Brown et al. (12) showed that regional injection with IL13Rα2-targeted CAR-T cells induced complete remission in a patient. Tchou et al. (13) suggested that intratumoral injections of CAR-T cells were safe and effective in breast tumors, and Priceman et al. (14) indicated that local intracranial delivery of HER2- CAR T cells was an effective method for targeting breast cancer brain metastasis in mice. Taken together, this evidence supports the local injection with CAR-T cell as a feasible method to eliminate the local tumor.

During the treatment process and follow-up, the local mass had a clear size reduction, and the remission status lasted for one year. In addition, no signs of severe adverse effects were observed, indicating intravenous infusion combined with local injection of CAR-T cell is an effective and safe strategy. Notably, the patient did not receive maintenance treatment after the CAR-T cell therapy; this suggests the specific efficacy is likely caused by CAR-T cell alone. A recent case report by Wei et al. (15) also suggests the combined strategy is an effective method for relapsed DLBCL patients; compared with that study, our study had a relatively long follow-up time, and the specific efficacy was certainly caused by CAR-T cell alone. Regardless, these findings suggest that intravenous infusion in combination with local injection of CAR-T cell is an effective and safe strategy in DLBCL patients with extranodal lesions.

The patient received an intravenous injection and local injection, it is hard to distinguish whether the elimination of the forearm lesion was caused solely by the local or systemic CAR-T cell therapy. In addition, it is difficult to compare the response at the different lesion sites because we did not conduct an imaging test to evaluate the status of the lesions before the patient achieved the CR. Nonetheless, we speculated the local injection played a more important role and showed a better response than the intravenous injection. The number of CAR-T cells infiltrating lesions was likely higher in local injection than the intravenous infusion, so our next research will elaborate on this. Moreover, the disease behavior for the patient is unusual, it may be caused by individual difference, and the individual difference may affect the results, so our team will enroll more patients.

In conclusion, our study suggests combining intravenous infusion with local injection is a feasible strategy for relapsed DLBCL patients with extranodal lesions. Our next plan will enroll more patients to determine the combined strategy’s efficacy and safety, explore the specific dose of local injectable CAR-T cells, and compare the local and systematic lesions’ response.

## Data Availability Statement

The raw data supporting the conclusions of this article will be made available by the authors, without undue reservation.

## Ethics Statement

The studies involving human participants were reviewed and approved by the Second Hospital of Anhui Medical University. The patients/participants provided their written informed consent to participate in this study. Written informed consent was obtained from the individual(s) for the publication of any potentially identifiable images or data included in this article.

## Author Contributions

All authors contributed to the article and approved the submitted version.

## Funding

This work was supported by the major subject of science and technology of Anhui province: [grant number 201903a07020030].

## Conflict of Interest

FY was employed by Jiangsu Tuohong Kangheng Pharmaceutical Co. Ltd.

The remaining authors declare that the research was conducted in the absence of any commercial or financial relationships that could be construed as a potential conflict of interest.

## Abbreviations

DLBCL, diffuse large B-cell lymphoma; CAR-T, Chimeric antigen receptor T cell; PET/CT, positron emission tomography-CT; FDP, fluoro-deoxy glucose; CRS, cytokine-release syndrome; CR, complete remission.
